# Isolation of casein for stable isotope ratio analysis of butter, cheese, and milk powder

**DOI:** 10.1002/rcm.9402

**Published:** 2023-01-02

**Authors:** Roisin O'Sullivan, Olaf Schmidt, Michael O'Sullivan, Raquel Cama‐Moncunill, Frank J. Monahan

**Affiliations:** ^1^ UCD School of Agriculture and Food Science University College Dublin Dublin Ireland

## Abstract

**Rationale:**

Stable isotope ratio analysis (SIRA) is commonly used for the authentication of dairy commodities, providing evidence to support the geographical origin and production background of products. We set out to optimise methods for the isolation of a common constituent (casein) from three dairy commodities, which would permit easier inter‐ and intra‐commodity comparisons following SIRA.

**Methods:**

Three published methods for isolation of protein (from cheese, milk, and butter) were adapted to yield protein (casein) fractions from commercial cheddar cheese, whole milk powder (WMP), and butter samples with a high degree of purity for subsequent SIRA. The casein fractions isolated underwent elemental analysis (H, C, and N), protein determination, and some also underwent SIRA of O and S. Two‐way analysis of variance and Tukey *post hoc* comparisons tested differences between methods.

**Results:**

For each product, an optimised casein isolation method was chosen based on the C/N ratio and protein content. An optimum solvent lipid extraction (petroleum spirit–diethyl ether (2:1)) and casein precipitation method was chosen for cheddar cheese casein. A final solvent lipid extraction (heptane–isopropanol (3:2)) was necessary for WMP and butter casein extraction. δ^13^C and δ^2^H values validated the methods' abilities to remove contaminating lipid and isolate pure casein.

**Conclusions:**

Casein of high purity, for subsequent SIRA, can be isolated from cheddar cheese, WMP, and butter following modifications of previously published methods.

## 1 INTRODUCTION

In the wake of conscious consumer behaviour following multiple high‐profile cases of food fraud, the authentication of food products is becoming increasingly important. Stable isotope ratio analysis (SIRA) for the authentication of milk,^1^ cheese,^2^ butter,^3^ and milk powders^4^ is a method of choice for many situations. In previous studies of milk and dairy products, cheese glycerol,^5^ milk^6^ or specific milk fractions including bulk milk powder,^7^ milk fat,^8^ milk water,^9^ lactose,^10^ or, most frequently, casein,^11^, ^12^, ^13^, ^14^ has been used for SIRA. In milk, using SIRA, δ^13^C and δ^15^N values have been measured in whole milk and isolated casein fractions,^5^, ^15^ while δ^18^O values have been measured in milk water fractions.^16^ In cheese, δ^13^C values were measured in the glycerol and casein fractions, δ^15^N and δ^34^S in the casein fraction only, and δ^18^O in the glycerol fraction only.^17^, ^18^

An advantage of using a specific fraction, such as casein, for comparisons of products, from different geographical origins or production systems for example, is that it eliminates the contribution of other fractions to variation in the stable isotope values between products. For example, the isotopic values of protein and lipid fractions differ substantially^12^, ^19^; therefore, differences in the isotopic values of whole dairy products could be due to differences in lipid concentration between the products as opposed to factors such as geographical origin or production parameters. A second advantage of using a protein fraction such as casein is that, because of its elemental composition, it is possible to measure the isotopic values of all the light elements common to foods (H, C, N, O, S); this is not the case with lipid fractions, in which N and S are limiting.

For milk samples, centrifugation has been commonly used to remove the lipid fraction, followed by acidification to pH 4.6 of the aqueous fraction to precipitate the casein fraction.^1^, ^5^, ^14^, ^20^ For cheese, the methods of Manca et al^21^ and Camin et al^2^ are often cited^22^, ^23^ for casein extraction while an adaptation of the method of Weber et al^24^ used in Camin et al^12^ is used for glycerol extraction.^17^, ^18^ For butter,^3^ bulk butter or butter protein obtained following solvent extraction of the lipid fraction has been used for SIRA. For milk powder, bulk whole milk powder (WMP)^25^ and bulk skimmed milk powder have been used.^4^ Thus, depending on the preparatory methods used, the proportions of lipid, protein, and other constituents may vary in the fractions used for SIRA, making it difficult to make comparisons between products based on SIRA data.

The objective of the study presented here was to develop methods for the isolation of a casein fraction of high purity from three different dairy products (cheddar cheese, WMP, and butter) that would enable subsequent intra‐ and inter‐product SIRA comparisons.

## 2 MATERIALS AND METHODS

### 2.1 Chemicals

Diethyl ether, isopropanol, sodium carbonate, and potassium sodium tartrate tetrahydrate were purchased from VWR Chemicals (Ballycoolin, Dublin). Petroleum spirit and Folin–Ciocalteau reagent were purchased from Sigma‐Aldrich (Wicklow, Ireland). Heptane was purchased from Lennox Laboratory Supplies (Dublin, Ireland). Hydrochloric acid (HCl; laboratory grade), casein (pure; product code 10545691), and sodium hydroxide (NaOH; analytical reagent grade) were purchased from Fisher Scientific (Loughborough, UK). Deionised water was obtained from a Millipore Elix 15 water purification system (Merck Millipore, Darmstadt, Germany). Cupric sulfate was purchased from BDH Chemicals Ltd (Poole, UK). Acetanilide standard was sourced from Exeter Analytical (Coventry, UK).

### 2.2 Isolation of casein fraction from cheddar cheese

**FIGURE 1:**
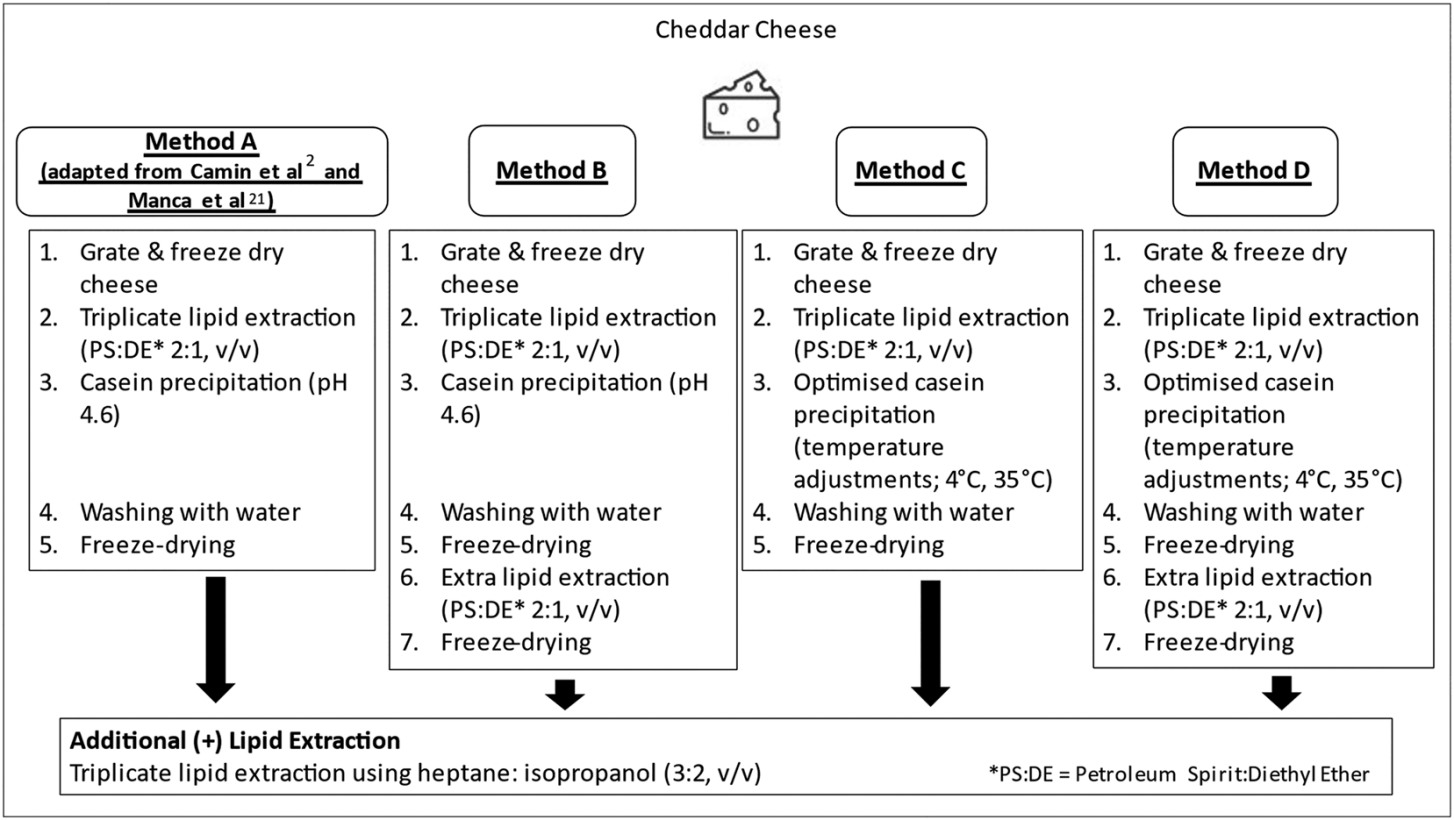
Summary of methods A, B, C, and D and of A+, B+, C+, and D+ (additional lipid extraction) for cheddar cheese casein isolation

The methods used to isolate the casein fraction from cheddar cheese are summarised in Figure 1. Each method was carried out in triplicate. In Method A (an adaptation of the methods of Camin et al^2^ and Manca et al^21^), grated cheese (6 g) was freeze‐dried (Buchi Lyovapor L‐200) for 24 h in 10 cm × 10 cm mini‐grip resealable sample bags (VWR, APHA121‐086). Using an Ultra‐Turrax (IKA DI 25 basic homogeniser; 13 500 rpm, 3 min), 4 g of the freeze‐dried cheese was homogenised with petroleum spirit–diethyl ether (2:1, v/v) (30 mL) in a 50 mL centrifuge tube (Corning®, 430828), centrifuged (Beckman Coulter Avanti J‐E centrifuge; 2500*g*, 6 min), and the supernatant was decanted. This step was repeated twice. The pellet was vortexed (Stuart Scientific SA8 vortex mixer) (30 s) with deionised water (30 mL) and acidified to pH 4.6 by dropwise addition of 1 M HCl using a pH meter (Mettler Toledo FE20/EL20). After gently agitating in a 50 mL glass beaker using a magnetic stir bar for 30 min, and centrifugation (1200*g*, 8 min), the supernatant was decanted. The casein pellet was suspended in 10 mL of deionised water, vortexed for 30 s, and centrifuged (1200*g*, 4 min). The water was decanted, and the remaining pellet was retained in a 6 cm × 6 cm sample bag and freeze‐dried. Freeze‐dried powder was stored in a desiccator before elemental (Section 2.6) and protein (Section 2.7) analysis and SIRA (Section 2.8).

In Method B the procedure outlined for Method A was followed with an additional lipid extraction step. Following the final freeze‐drying step, the resulting casein powder was homogenised using an Ultra‐Turrax (13 500 rpm, 1 min) with 10 mL of petroleum spirit–diethyl ether (2:1, v/v) in a 50 mL centrifuge tube, centrifuged (2500*g*, 6 min), and the supernatant was decanted. The remaining casein pellet was placed into a 6 cm × 6 cm open sample bag and freeze‐dried. Freeze‐dried powder was stored in a desiccator before elemental (Section 2.6) and protein (Section 2.7) analysis and SIRA (Section 2.8).

In Method C the procedure outlined in Method A was following with additional temperature adjustments to improve final casein yield.^26^ Prior to acidification, the pellet was cooled to approx. 4°C in a refrigerator and, after acidification, was held in a water bath at approx. 35°C for at least 30 min.

In Method D the procedure outlined for Method C was followed with an additional lipid extraction step. Following the final freeze‐drying step, the resulting casein powder was homogenised using an Ultra‐Turrax (13 500 rpm, 1 min) with 10 mL of petroleum spirit–diethyl ether (2:1, v/v) in a 50 mL centrifuge tube, centrifuged (2500*g*, 6 min), and the supernatant was decanted. The remaining casein pellet was placed into a 6 cm × 6 cm open sample bag and freeze‐dried. Freeze‐dried powder was stored in a desiccator before elemental (Section 2.6) and protein (Section 2.7) analysis and SIRA (Section 2.8).

### 2.3 Isolation of casein fraction from WMP

**FIGURE 2:**
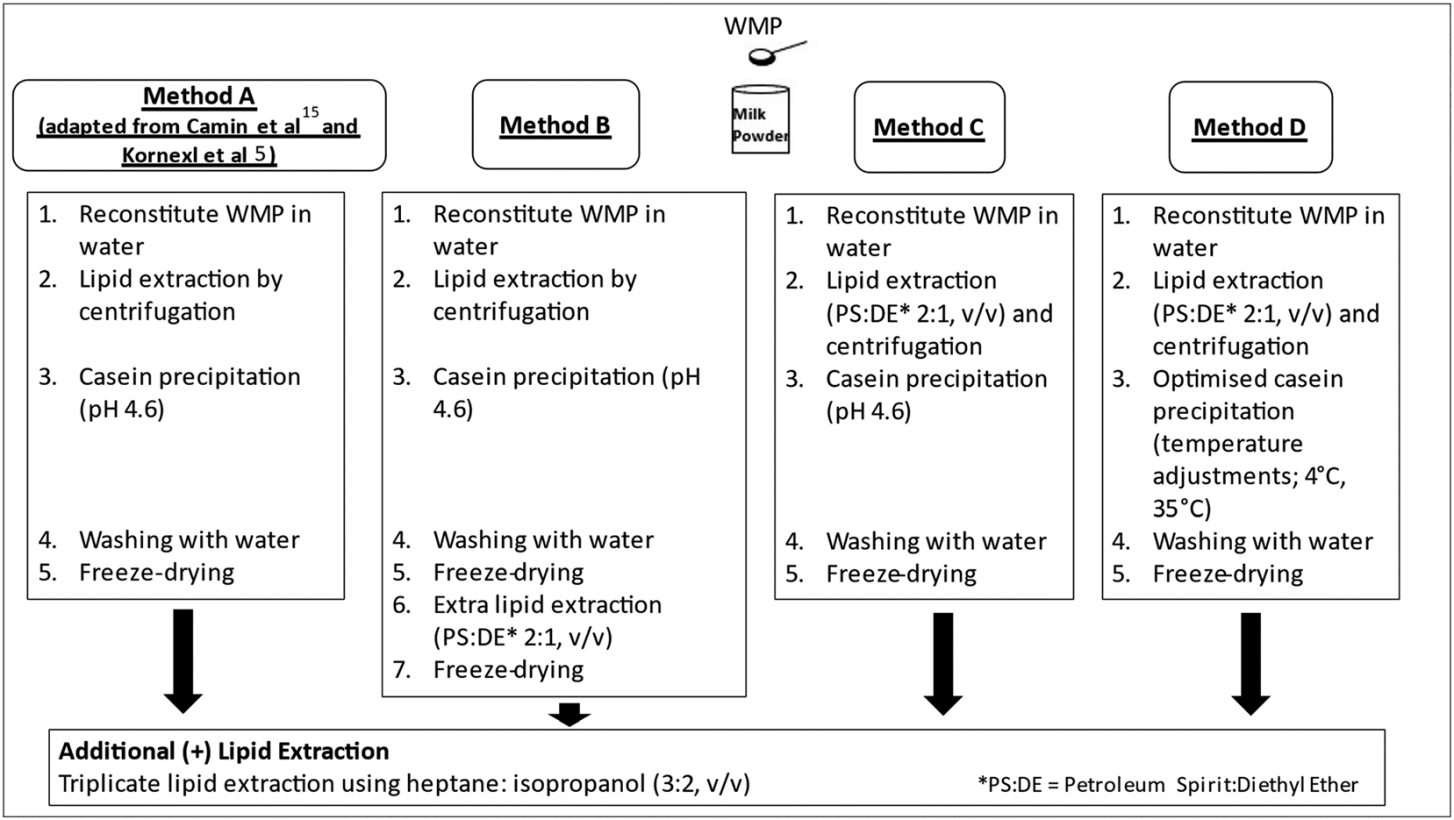
Summary of methods A, B, C, and D and of A+, B+, C+, and D+ (additional lipid extraction) for WMP casein isolation

The methods used to isolate the casein fraction from WMP are summarised in Figure 2. Each method was carried out in triplicate. Previous studies conducting SIRA of milk powders have used the bulk milk powder rather than casein isolated from the milk powder.^4^, ^25^, ^27^ Therefore, Method A was adapted from the methods in Camin et al^15^ and Kornexl et al^5^ for casein isolation from liquid milk. WMP (1 g) was reconstituted in deionised water (10 mL) in a 15 mL centrifuge tube (Kartell, KART84000) and placed in a shaking water bath (Grant Instruments GLS400 linear shaking water bath) at room temperature for 30 min. The sample was centrifuged (5000*g*, 15 min) and the upper lipid layer removed using a transfer pipette. The lower, aqueous layer was acidified to pH 4.6 by dropwise addition of 1 M HCl. After holding for 2 min and centrifugation (5400*g*, 10 min) the supernatant was removed. The casein pellet was resuspended in 10 mL of deionised water, vortexed for 30 s, and centrifuged (5400*g*, 2 min). The aqueous supernatant was removed, and the washed casein pellet was placed in an open 6 cm × 6 cm sample bag and freeze‐dried. Freeze‐dried powder was stored in a desiccator before elemental (Section 2.6) and protein (Section 2.7) analysis and SIRA (Section 2.8).

In Method B the procedure outlined for Method A was followed with an additional lipid extraction step. Following the final freeze‐drying step, the resulting casein powder was weighed into a 50 mL glass beaker and reconstituted using a magnetic stir bar with 10 mL of warm water (approx. 40–50°C) for a minimum of 1 h. Petroleum spirit–diethyl ether (2:1, v/v) (10 mL) was added and left to stir for a further minimum of 15 min. Following centrifugation (5000*g*, 15 min), the supernatant was decanted, and the defatted casein pellet was placed in an open 6 cm × 6 cm sample bag and freeze‐dried. Freeze‐dried powder was stored in a desiccator before elemental (Section 2.6) and protein (Section 2.7) analysis and SIRA (Section 2.8).

In Method C the procedure outlined for Method A was followed with an additional solvent lipid extraction step. Petroleum spirit–diethyl ether (2:1, v/v) (10 mL) was added to reconstituted WMP which was agitated for 15 min (in a 50 mL glass beaker using a stir bar and magnetic stir plate) prior to centrifugation (5000*g*, 15 min) and removal of the upper lipid layer.

In Method D the procedure outlined in Method C was followed with additional temperature adjustments to improve final casein yield.^26^ Prior to acidification, the pellet was cooled to approx. 4°C in a refrigerator and, after acidification, was held in a water bath at approx. 35°C for at least 30 min.

### 2.4 Isolation of casein fraction from butter

**FIGURE 3:**
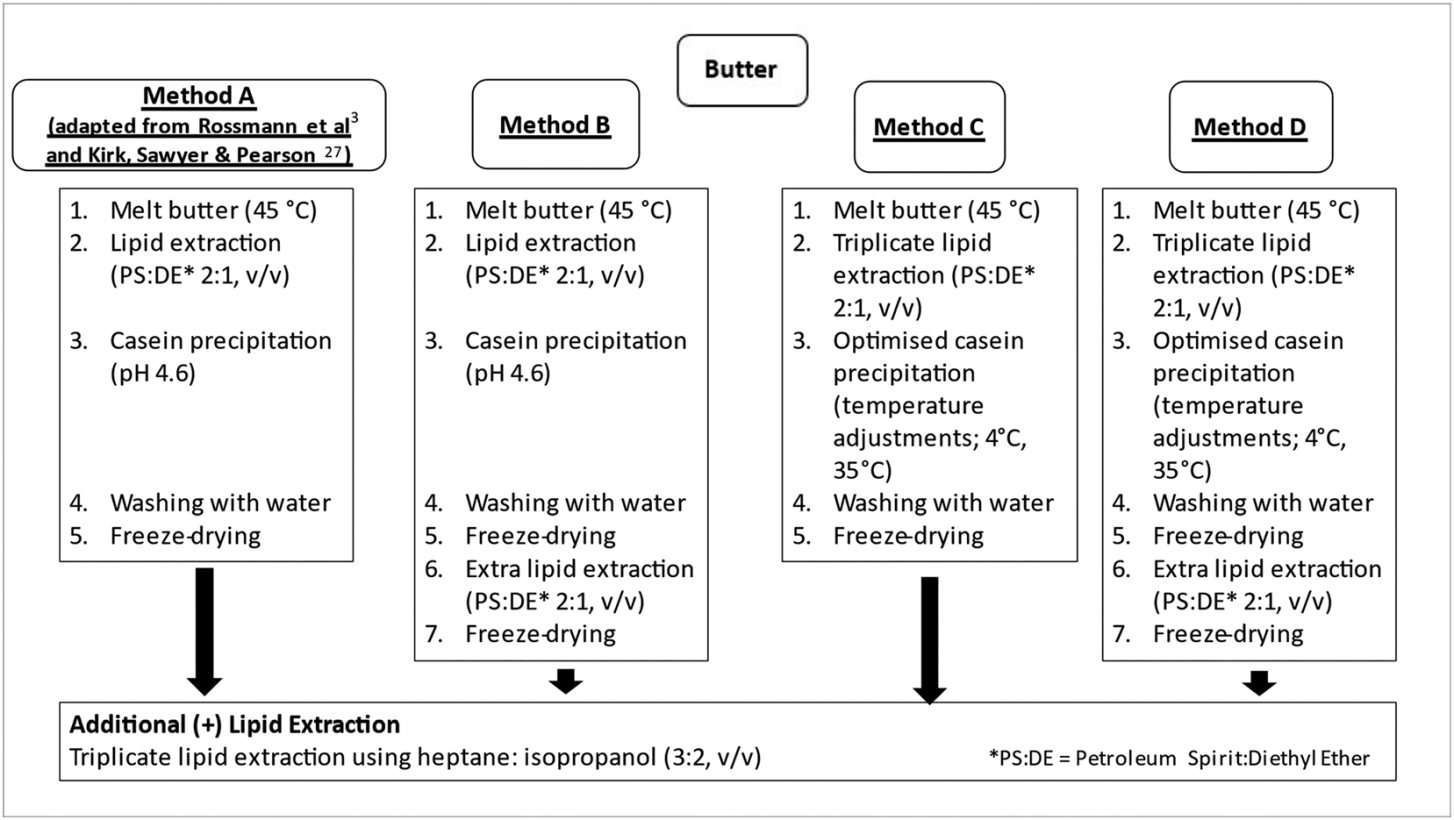
Summary of methods A, B, C, and D and of A+, B+, C+, and D+ (additional lipid extraction) for butter casein isolation

The methods applied to the isolation of casein from butter are summarised in Figure 3. Each method was carried out in triplicate. In Method A (adaptation of the methods in Rossmann et al^3^ and Kirk et al^26^), butter (30 g) in a 250 mL centrifuge tube (Nalgene PPCO) was melted in a shaking water bath at approx. 40°C for 30 min. Petroleum spirit–diethyl ether (2:1, v/v) (150 mL) was added to the melted butter in 3 × 50 mL volumes using a glass pipette (50 mL) which was held in a fume hood for 30 min. In a 250 mL separating funnel (DWK Life Sciences Limited, 2180/06M) the aqueous and lipid phases were separated, and the lower aqueous phase was collected in a 50 mL centrifuge tube (using 10 mL of deionised water to improve separation and 10 mL of petroleum spirit–diethyl ether (2:1, v/v) to rinse). The aqueous phase was acidified to pH 4.6 by dropwise addition of 1 M HCl. After gentle agitation for 30 min in a shaking water bath at room temperature, the contents were centrifuged (1200*g*, 20 min) and the supernatant was decanted. The casein pellet was vortexed (30 s) with 10 mL of deionised water, centrifuged (1200*g*, 20 min), and the washed casein pellet was placed into an open 6 cm × 6 cm sample bag and freeze‐dried. Freeze‐dried powder was stored in a desiccator before elemental (Section 2.6) and protein (Section 2.7) analysis and SIRA (Section 2.8).

In Method B the procedure outlined for Method A was followed with an additional lipid extraction step. Following the final freeze‐drying step, the resulting casein powder was weighed into a 50 mL centrifuge tube with 30 mL of petroleum spirit–diethyl ether (2:1, v/v), placed in a shaking water bath at room temperature for approx. 30 min, and centrifuged (1200*g*, 20 min). The supernatant was removed, and the washed casein was placed into a 6 cm × 6 cm sample bag and freeze‐dried. Freeze‐dried powder was stored in a desiccator before elemental (Section 2.6) and protein (Section 2.7) analysis and SIRA (Section 2.8).

In Method C the procedure outlined for Method A was followed with an additional lipid extraction step and additional temperature adjustments to improve final casein yield.^26^ Petroleum spirit–diethyl ether (2:1, v/v) (30 mL) was added to the aqueous phase following separation in the separating funnel, centrifuged (1200*g*, 10 min), and the supernatant was decanted. This step was repeated with 15 mL of petroleum spirit–diethyl ether (2:1, v/v) (15 mL). The aqueous layer was then cooled to approx. 4°C in a refrigerator before acidification and after which it was held in a water bath at approx. 35°C for at least 30 min.

In Method D the procedure outlined for Method C was followed with an additional lipid extraction step. Following the final freeze‐drying step, the resulting casein powder was weighed into a 15 mL centrifuge tube with 10 mL of petroleum spirit–diethyl ether (2:1, v/v), placed in a shaking water bath at room temperature for approx. 30 min, and centrifuged (1200*g*, 20 min). The supernatant was removed, and the washed casein was placed into a 6 cm × 6 cm sample bag and freeze‐dried. Freeze‐dried powder was stored in a desiccator before elemental (Section 2.6) and protein (Section 2.7) analysis and SIRA (Section 2.8).

### 2.5 Additional heptane–isopropanol (3:2, v/v) extraction

As a result of the data obtained following the elemental and protein analyses of casein fractions from cheese, WMP, and butter following methods Methods A–D above, an additional lipid extraction step, using a more polar solvent (heptane–isopropanol (3:2, v/v)), was applied. This additional modification was based in the procedure described in Radin,^28^ although hexane was replaced with heptane for safety reasons.^29^

The freeze‐dried cheese, WMP, and butter casein fractions collected following Methods A, B, C, and D were transferred from sample bags to 50 mL centrifuge tubes and 5 mL of heptane–isopropanol (3:2, v/v) was added. The suspension was homogenised using an Ultra‐Turrax (9500 rpm for 1 min) and centrifuged (5400*g*, 10 min) at room temperature (approx. 20°C). The supernatant was decanted and a further 2.5 mL of heptane–isopropanol (3:2, v/v) was added, followed by homogenisation, centrifugation, and removal of the supernatant. This step was repeated once again, and any remaining solvent was evaporated from the defatted casein by leaving in a fume hood overnight. The casein fraction was collected and prepared for elemental (Section 2.6) and protein (Section 2.7) analysis and SIRA (Section 2.8).

### 2.6 Elemental analysis

Hydrogen, carbon, and nitrogen analysis was conducted with an Exeter CE440 CHN analyser. The standard used was acetanilide. Each sample (1.6–1.9 mg) was weighed on a Sartorius Cubis MSE 2.7S‐000‐DM Ultra microbalance into a tin capsule and introduced via a ladle to a pure oxygen environment in the combustion tube. After combustion the sample residue was removed and the mixture of combustion products (CO_2_, H_2_O, and N_2_) pulsed into a mixing chamber at constant temperature and pressure. A known volume of the product mixture was released when a pre‐set pressure (e.g. 1500 mmHg) was reached. The mixture then passed through a series of traps where H_2_O and CO_2_ were completely absorbed, with high precision thermal conductivity detector filaments located before and after each absorption trap. The difference between the output of each set of detectors before and after absorption is proportional to the trapped component and hence the quantity of carbon and hydrogen in the original sample can be determined. Nitrogen is measured with reference to pure helium carrier gas, the difference in thermal conductivity being proportional to nitrogen content. Typically, the precision is ±0.3% for homogeneous, stable, solid samples.

### 2.7 Protein determination

Protein concentration was determined following the Lowry method^30^ using pure casein (Section 2.1) as a standard. Standard and samples were prepared in 0.1M NaOH. The procedure involved the addition of 0.1 mL 1M NaOH, 5 mL Biuret reagent (prepared by mixing an alkaline sodium carbonate solution with a copper sulfate solution) and 0.5 mL dilute Folin‐Ciocalteau reagent to 1 mL of standard or sample solution and a hold time of 30 min at room temperature, followed by measurement of absorbance at 740nm on a UV‐Vis spectrophotometer (Shimadzu, UVmini‐1240). The percentage protein was calculated based on the pure casein standard calibration curve.

### 2.8 Stable isotope ratio analysis

All samples were analysed at Iso‐Analytical Ltd. (Crewe, UK) by Elemental‐Analysis – Isotope Ratio Mass Spectrometry (EA‐IRMS), using a Europa Scientific elemental analyser and 20–20 IRMS. Stable Isotope Ratio Analysis (SIRA) of C, N, and S and O was conducted according to methods described in detail in O'Sullivan et al.^14^ Briefly, carbon dioxide gas and nitrogen gas were measured simultaneously for δ^13^C and δ^15^N values, while sulphur dioxide gas were measured separately for δ^34^S. For δ^18^O, samples along with calibration standards were comparatively equilibrated for 7 days prior to analysis and carbon monoxide and nitrogen gases were separated on a GC column packed with a molecular sieve at 45 °C. The analysis followed a batch process by which a reference was analysed followed by a number of samples and then another reference. The main standard reference materials used were soy protein (δ^13^C_V‐PDB_ = −25.22 ‰, δ^15^N_AIR_ = 0.99 ‰), barium sulphate (δ^34^S_V‐CDT_ = +20.33 ‰), and cane sugar (δ^18^O_V‐SMOW_ = 35.23 ‰) for δ^13^C, δ^15^N, δ^34^S, and δ^18^O, respectively. These standards are calibrated and traceable to IAEA inter‐laboratory standards. In addition, check samples were run during batch analysis for quality control purposes. Based on these, the analytical precision (SD) in the present study was 0.02 ‰ for δ^13^C (soy protein), 0.01 ‰ for δ^15^N (soy protein), 0.06 ‰ for δ^34^S (barium sulphate) and 0.23 ‰ for δ^18^O (cane sugar).

For δ^2^H, samples and references were weighed (1.0 ± 0.1 mg) into silver capsules and comparatively equilibrated with moisture in the laboratory air for 14 days prior to analysis. Five non‐exchangeable hydrogen standards were comparatively equilibrated and analysed alongside samples as controls. Of these, three standards used for calibration were human hair (non‐exchangeable δ^2^H_V‐SMOW_ = −44.4 ‰), caribou hoof (non‐exchangeable δ^2^H_V‐SMOW_ = −157.0 ‰) and kudu horn (non‐exchangeable δ^2^H_V‐SMOW_ = ‐35.3 ‰). The further two standards, measured as quality control check samples, were human hair (non‐exchangeable δ^2^H_V‐SMOW_ = −72.9 ‰) and casein (non‐exchangeable δ^2^H_V‐SMOW_ = −113.37 ‰). Analytical precision was 0.45 ‰ for δ^2^H (mineral oil).

### 2.9 Statistical analysis

The effect of the use of different methods of casein extraction on the C/N ratio, protein (%) and stable isotope ratio values of the products was tested using a two‐way analysis of variance (ANOVA) followed by a Tukey test for post‐hoc comparisons. The statistical analysis of the data was performed using R^31^ and the packages used were *car* ^38^ (test of homogeneity of variances), *stats* ^31^ (ANOVA), *agricolae* ^39^ (Tukey post hoc test) and *dplyr* ^40^ (data manipulation).

## 3 RESULTS AND DISCUSSION

The three products analysed in this study (cheddar cheese, WMP, and butter) have undergone various levels of processing during manufacture and may be described as complex food matrices. Isolating a common constituent (casein) from each is therefore useful in permitting stable isotope ratio comparisons both within and between product categories. However, differences in the products' compositions and manufacturing methods pose challenges in terms of extraction of the casein fraction because of the potential for interference from other constituents, most notably lipid. Milk fat constitutes a minimum (on dry matter basis) of 22% of cheddar cheese,^32^ 26% of WMP,^33^ and 80% of butter.^34^ However, a further challenge is the low casein content of butter, with casein estimated to be about 0.48% by fresh weight of butter. Therefore, extraction methods which optimise lipid removal and yield a casein fraction of high purity, following extraction, are desirable.

A positive linear relationship between C/N ratio values and lipid content has been recorded in muscle tissue samples.^35^ The C/N ratio for milk casein is approximately 3.5,^14^ with higher values being indicative of interference from lipid. For this reason, elemental analysis together with protein determination were used to evaluate the purity of the casein fractions extracted from cheese, WMP, and butter using the various methods outlined in Section 2.1.

### 3.1 Cheddar cheese: elemental and protein analyses of casein fraction

The C/N ratio of casein extracted from cheddar cheese ranged from 3.50 to 3.55 (Table 1) and was consistent across the various extraction methods (*p* < 0.05). The ratio was also in agreement with that of commercial casein (3.45) (Fisher Scientific, product code 10545691) which was determined by the same elemental analysis method (Section 2.1). The protein content of the casein fractions obtained across all methods, measured against the commercial casein standard, was close to 100% (Table 1) (*p* < 0.05). Based on these data, Method A, being the most straightforward, was selected as the method of choice for casein isolation from cheddar cheese for subsequent SIRA.

**TABLE 1:** Hydrogen, carbon, and nitrogen content, C/N ratio, protein content, and isotope ratio values of cheese casein obtained following each of methods A, B, C, and D alone or with an additional heptane–isopropanol extraction step (methods A+, B+, C+, D+)

Method	H (±SD), %	C (±SD), %	N (±SD), %	C/N (±SD)	Protein (±SD), %	δ^15^N (±SD), ‰	δ^13^C (±SD), ‰	δ^34^S (±SD), ‰	δ^2^H (±SD), ‰	δ^18^O (±SD), ‰
A	6.17 (± 0.24)	42.91 (± 2.27)	12.08 (± 0.69)	3.55 (± 0.01)^a^	105.22 (± 3.44)^a^	7.21 (± 0.02)^a^	−27.16 (± 0.02)^a^	6.43 (± 0.01)^c^	−108.48 (± 0.09)^b^	16.46 (± 0.23)^a^
A+	6.65 (± 0.05)	47.07 (± 0.51)	13.42 (± 0.26)	3.51(± 0.04)^a^	108.22 (± 1.81)^a^					
B	6.01 (± 0.06)	42.02 (± 0.63)	11.84 (± 0.16)	3.55 (± 0.01)^a^	107.09 (± 2.04)^a^					
B+	6.68 (± 0.09)	47.55 (± 0.48)	13.52 (± 0.12)	3.52 (± 0.04)^a^	108.87 (± 1.23)^a^	7.16 (± 0.02)^ab^	−27.23 (± 0.06)^a^	6.88 (± 0.10)^b^	−108.07 (± 1.49)^b^	14.69 (± 0.01)^b^
C	6.13 (± 0.25)	42.29 (± 0.70)	11.94 (± 0.21)	3.54 (± 0.01)^a^	108.27 (± 2.32)^a^					
C+	6.34 (± 0.18)	45.71 (± 1.60)	12.93 (± 0.70)	3.54 (± 0.07)^a^	104.95 (± 4.29)^a^	7.12 (± 0.02)^b^	−27.22 (± 0.01)^a^	7.13 (± 0.03)^a^	−101.58 (± 1.01)^a^	13.93 (± 0.31)^c^
D	6.94 (± 0.07)	47.55 (± 0.43)	13.49 (± 0.13)	3.53 (± 0.01)^a^	110.74 (± 16.07)^a^					
D+	6.44 (± 0.19)	47.17 (± 0.15)	13.47 (± 0.06)	3.50 (± 0.01)^a^	107.66 (± 2.25)^a^					

^a,b,c^ Values in a column with common superscripts are not significantly different (Tukey, *p* > 0.05).

### 3.2 WMP: elemental and protein analyses of casein fraction

The C/N ratio of casein extracted from WMP varied widely across all methods (range 3.56 to 9.29) (Table 2). At the higher C/N ratio, the lower protein content of the extracted fractions suggested possible contamination with lipid. The additional lipid extraction step using heptane–isopropanol led to increased purity of the casein isolate (*p* > 0.05) particularly for Methods A and B (Table 2). Based on these data, Method B+, having a C/N ratio (3.56) closest to that of commercial casein (3.45) (Fisher Scientific, product code 10545691) and yielding the highest protein content (103.1%), was selected as the method of choice for casein isolation from WMP for subsequent SIRA.

**TABLE 2:** Hydrogen, carbon, and nitrogen content, C/N ratio, protein content, and isotope ratio values of WMP casein obtained following each of methods A, B, C, and D alone or with an additional heptane–isopropanol extraction step (methods A+, B+, C+, D+)

Method	H (±SD), %	C (±SD), %	N (±SD), %	C/N (±SD)	Protein (±SD), %	δ^15^N (±SD), ‰	δ^13^C (±SD), ‰	δ^34^S (±SD), ‰	δ^2^H (±SD), ‰	δ^18^O (±SD), ‰
A	8.48 (± 0.27)	56.88 (± 1.28)	7.18 (± 0.11)	7.93 (± 0.25)^ab^	57.79 (± 5.42)^b^	6.34 (± 0.17)^a^	−28.55 (± 0.03)^b^	4.81 (± 0.08)^b^	−164.52 (± 11.81)^b^	16.47 (± 0.86)^a^
A+	6.62 (± 0.07)	46.76 (± 0.16)	12.35 (± 0.24)	3.79 (± 0.07)^b^	101.12 (± 3.91)^a^					
B	8.81 (± 0.01)	58.16 (± 0.30)	7.26 (± 0.10)	8.01 (± 0.11)^ab^	55.18 (± 0.84)^b^					
B+	6.65 (± 0.06)	47.81 (± 0.20)	13.44 (± 0.11)	3.56 (± 0.04)^b^	103.06 (± 2.37)^a^	6.43 (± 0.06)^a^	−26.51 (± 0.17)^a^	5.13 (± 0.05)^a^	−107.06 (± 1.26)^a^	14.96 (± 0.34)^a^
C	6.21 (± 0.14)	40.05 (± 1.93)	4.43 (± 1.31)	9.05 (± 2.24)^a^	37.83 (± 2.14)^b^					
C+	6.65 (± 0.09)	45.48 (± 0.27)	9.51 (± 1.06)	4.78 (± 0.60)^b^	83.04 (± 12.50)^a^	6.38 (± 0.07)^a^	−26.59 (± 0.07)^a^	4.55 (± 0.08)^c^	−104.19 (± 3.19)^a^	15.47 (± 0.37)^a^
D	7.83 (± 1.16)	50.98 (± 7.54)	5.49 (± 1.31)	9.29 (± 2.97)^a^	44.19 (± 7.08)^b^					
D+	6.80 (± 0.06)	47.19 (± 0.06)	11.75 (± 0.22)	4.02 (± 0.07)^b^	96.44 (± 9.23)^a^					

^a,b,c^ Values in a column with common superscripts are not significantly different (Tukey, *p* > 0.05).

### 3.3 Butter: elemental and protein analyses of casein fraction

Butter C/N ratios varied across isolation methods (range 3.71 to 7.58) and lower protein contents were associated with higher C/N ratios (Table 3). The additional heptane–isopropanol extraction increased the purity of the casein isolates (*p* > 0.05). Based on these data, Method B+, having a C/N ratio (3.71) closest to that of standard casein and yielding the highest protein content (91.1%), was selected as the method of choice for casein isolation from butter for subsequent SIRA.

**TABLE 3:** Hydrogen, carbon, and nitrogen content, C/N ratio, protein content, and isotope ratio values of butter casein obtained following each of methods A, B, C, and D alone or with an additional heptane–isopropanol extraction step (methods A+, B+, C+, D+)

Method	H (±SD), %	C (±SD), %	N (±SD), %	C/N (±SD)	Protein (±SD), %	δ^15^N (±SD), ‰	δ^13^C (±SD), ‰	δ^34^S (±SD), ‰	δ^2^H (±SD), ‰	δ^18^O (±SD), ‰
A	8.41 (± 0.13)	55.17 (± 0.21)	7.27 (± 0.15)	7.58 (± 0.17)^a^	41.62 (± 4.14)^e^	6.42 (± 0.12)^a^	−28.17 (± 0.22)^b^	5.99 (± 0.07)^b^	−184.81 (± 12.60)^b^	16.52 (± 0.33)^a^
A+	6.14 (± 0.10)	43.44 (± 0.26)	11.50 (± 0.13)	3.78 (± 0.04)^d^	75.80 (± 1.75)^bc^					
B	6.92(± 0.50)	47.18 (± 2.65)	9. 24 (± 0.63)	5.11 (± 0.63)^bc^	70.29 (± 8.33)^bcd^					
B+	5.56 (± 0.44)	39.64 (± 3.08)	10.68 (± 1.23)	3.71 (± 0.16)^d^	93.95 (± 4.10)^a^	6.39 (± 0.04)^a^	−25.29 (± 0.13)^a^	6.33 (± 0.02)^a^	−97.36 (± 0.43)^a^	15.69 (± 0.27)^ab^
C	6.27 (±0.51)	42.08 (± 3.69)	6.94 (± 0.98)	6.07 (± 0.32)^b^	56.20 (± 0.89)^de^					
C+	5.16 (± 0.44)	36.73 (± 3.16)	9.34 (± 1.12)	3.93 (± 0.13)^d^	76.24 (± 3.02)^bc^	6.11 (± 0.002)^b^	−25.41 (± 0.11)^a^	6.43 (± 0.04)^a^	−82.43 (± 2.42)^a^	15.23 (± 0.59)^b^
D	4.81 (± 0.58)	32.79 (± 4.08)	6.55 (± 1.21)	5.00 (± 0.37)^c^	62.18 (± 6.97)^cd^					
D+	4.33 (± 0.50)	30.16 (± 3.55)	7.04 (± 1.43)	4.28 (± 0.34)^cd^	79.67 (± 0.20)^ab^					

^a,b,c,d,e^ Values in a column with common superscripts are not significantly different (Tukey, *p* > 0.05).

### 3.4 SIRA of casein fractions isolated using different casein isolation methods

SIRA of H, C, N, O, and S was undertaken to provide further insight into potential effects of the presence of lipid in proteinaceous fractions on isotope data. Lipids are known particularly to be depleted in ^2^H and ^13^C isotopes compared to proteinaceous tissues, resulting in more negative δ^13^C and δ^2^H values.^36^, ^37^ Therefore, for the purposes of this discussion, the focus will be on δ^13^C and δ^2^H values. Evidence of the effect of casein purity and lipid interference on SIRA data was obtained following SIRA of casein fractions obtained following Methods A, B+, and C+ across all products. Method A was chosen as the most direct and least time‐consuming. Used with cheese, it also yielded a high protein casein fraction with an acceptable C/N ratio. Method B+ was chosen because it yielded a high protein casein fraction with acceptable C/N ratio from WMP and butter. Method C+ was chosen because it yielded a casein fraction with an intermediate level of purity and C/N ratio from WMP and butter. For cheddar cheese (Table 1) the SIRA data show agreement in δ^13^C and δ^2^H values across the three methods supporting the data indicating a high degree of purity in the casein fraction and a low level of lipid interference for all methods. For WMP (Table 2), the more negative δ^13^C and δ^2^H values for casein extraction with Method A (*p* > 0.05) is consistent with a higher lipid content while Methods B+ and C+ yield values consistent with a casein fraction of higher purity and lower lipid interference. The data justify Method B+ as the method of choice for WMP. For butter, the more negative δ^13^C and δ^2^H values for casein extraction with Method A (*p* > 0.05) is consistent with a higher lipid content. Similar to WMP, the data justify Method B+ as the method of choice for butter.

## 4 CONCLUSIONS

Through the systematic adoption of a number of approaches, involving centrifugation, pH and temperature adjustment, and solvent extraction, it is possible to obtain from different dairy products a casein fraction with a high degree of purity suitable for SIRA. The approaches adopted are more complex, requiring additional solvent extraction, in the case of WMP and butter. The advantage of isolating a casein fraction with a high degree of purity lies in the potential to make meaningful inter‐ and intra‐product comparisons of the effects of geographical origin, animal production system, or processing on stable isotope composition and to provide evidence to support authenticity claims.

### PEER REVIEW

The peer review history for this article is available at https://publons.com/publon/10.1002/rcm.9402.

## Data Availability

The data that support the findings of this study are available from the corresponding author upon reasonable request.
